# Novel qEEG Biomarker to Distinguish Anti-NMDAR Encephalitis From Other Types of Autoimmune Encephalitis

**DOI:** 10.3389/fimmu.2022.845272

**Published:** 2022-02-15

**Authors:** Tomotaka Mizoguchi, Makoto Hara, Satoshi Hirose, Hideto Nakajima

**Affiliations:** Division of Neurology, Department of Medicine, Nihon University School of Medicine, Tokyo, Japan

**Keywords:** anti-*N*-methyl-d-aspartate receptor encephalitis, autoimmune encephalitis (AE), quantitative electroencephalogram (qEEG), biomarker, diagnosis

## Abstract

**Objective:**

To establish the diagnostic biomarker of electroencephalogram (EEG) to distinguish between anti-*N*-methyl-d-aspartate receptor encephalitis (NMDARE) and other types of autoimmune encephalitis (other AEs).

**Methods:**

We reviewed the clinical records of 90 patients with acute encephalitis who were treated in our institution between January 2014 and October 2020. We enrolled the patients who fulfilled the diagnostic criteria for possible AE (pAE) defined by Graus et al. (pAE criteria) and then classified into definite NMDARE and other AEs. We investigated the main syndrome and analyzed all admission EEGs using EEG power value (PV). Statistical significance was tested using the Mann–Whitney *U* test or Fisher’s exact test.

**Results:**

Twenty-five patients fulfilled the pAE criteria and were classified into 9 with definite NMDARE (median age: 21 years; 8 women) and 12 with other AEs (median age: 37.5 years; 6 women). Four were eventually excluded. Speech dysfunction (9/9 vs. 4/12, *p* = 0.005) and movement disorders (6/9 vs. 1/12, *p* = 0.016) were more frequent in NMDARE than in other AEs. The PV analyses revealed the novel quantitative EEG (qEEG) index, namely, fast slow ratio (FSR) (PV of total beta/PV of total theta + delta). The median FSR (0.139 vs. 0.029, *p* = 0.004) was higher for NMDARE than other AEs, and the receiver operating characteristic curve area of FSR was 0.86 (95% CI 0.70–1.00). A cutoff value of 0.047 yielded a specificity of 0.75 and a sensitivity of 1.00. Focusing on patients who did not meet the “probable NMDARE criteria” in Graus 2016 (proNMDARE criteria) (*n* = 10), the pretest probability of NMDAR antibody test was 0.30 (3/10), which increased in patients with an FSR greater than the cutoff (*n* = 5) to 0.60 (3/5).

**Conclusions:**

The NMDARE group highlighted speech dysfunction and movement disorders, and a novel qEEG index FSR accurately distinguished the NMDARE patients from other AEs. The FSR is a promising diagnostic marker for NMDARE that indicates the positive results of NMDAR antibodies in patients with AE when combined with the proNMDARE criteria.

## Introduction

Antibodies against anti-*N*-methyl-d-aspartate receptor (NMDAR) trigger anti-*N*-methyl-d-aspartate receptor encephalitis (NMDARE), a well-characterized autoimmune encephalitis (AE) whose features include psychiatric symptoms, seizures, decreased level of consciousness, movement disorders, autonomic disabilities, and hypoventilation (1, 2). Early immunotherapies and/or removal of the associated tumor are key to favorable outcomes in NMDARE (3). However, physicians still struggle to identify NMDAR antibodies soon enough to best treat the disease. Graus et al. developed syndrome-based diagnostic criteria of probable NMDARE (proNMDARE) available without any antibody test (4), but their sensitivity was deemed unsatisfactory in the first 2 weeks of disease onset (5). These limitations prompted researchers to explore diagnostic biomarkers that distinguished NMDARE from other types of AE (other AEs) in early stages, including CSF cytokines, ^18^F-FDG PET, resting-state functional magnetic resonance imaging (MRI), and electroencephalogram (EEG) (6).

Recent analyses of EEG revealed that extreme delta brush (EDB) is highly specific for the patients with severe NMDARE (7). EDB consists of rhythmic beta activity overlying the rhythmic delta activity. Other EEG characteristics on NMDARE such as excessive beta activity and generalized rhythmic delta activity (GRDA) were also reported (8). These features could be used to non-invasively distinguish NMDARE from other AEs, though the sensitivity of EDB is approximately 30% as described in the first report (7).

The aim of the present study is to establish a novel index of quantitative EEG (qEEG) by using power value (PV) analysis and validate its ability to distinguish NMDARE from other AEs.

## Materials and Methods

### Protocol Approval and Patient Classification

**Figure 1:**
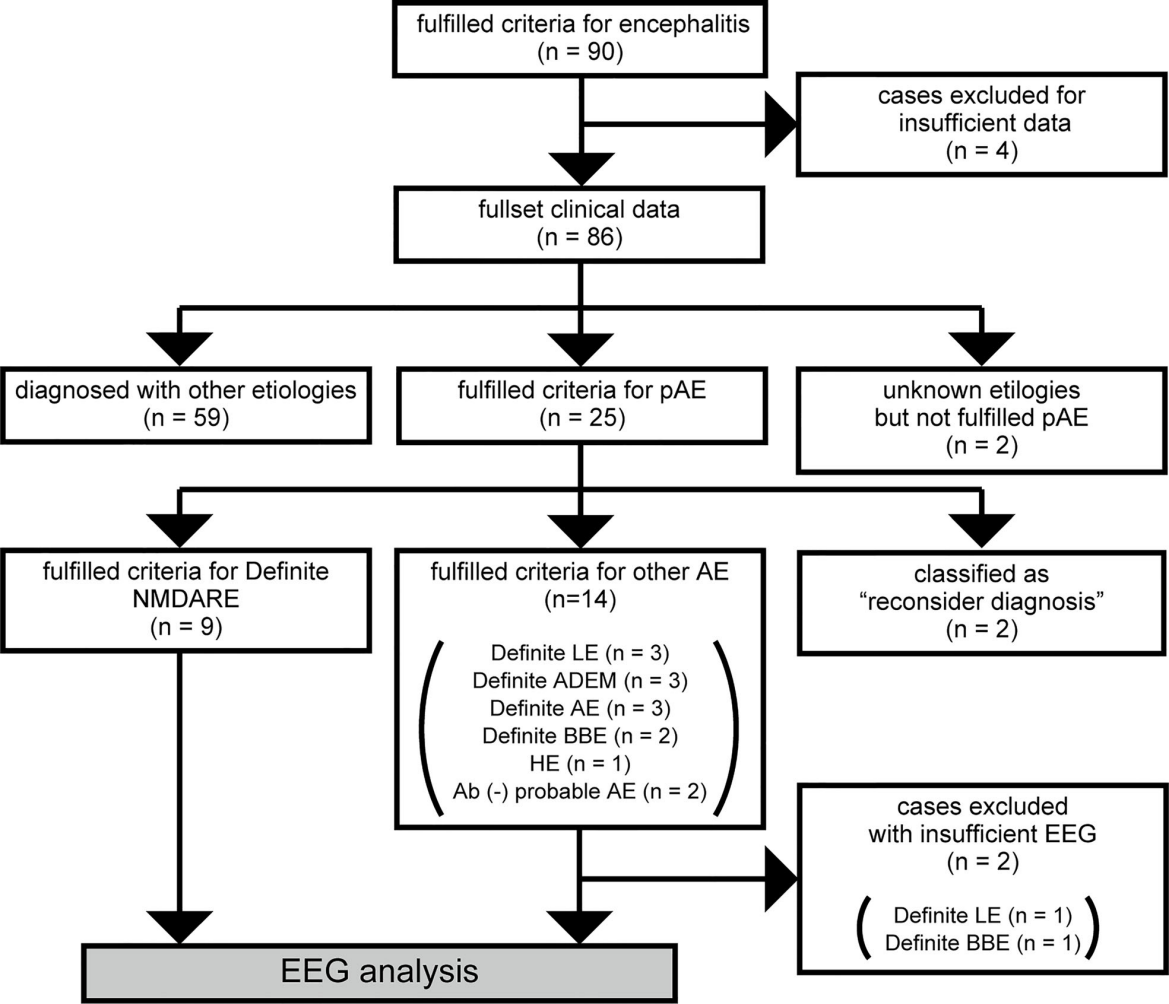
Flowchart of patient selection and classification. Out of 90 cases that fulfilled diagnostic criteria for acute encephalitis, four were excluded because of insufficient clinical data. Out of the other 86 cases, 59 were diagnosed with encephalitis of etiologies other than autoimmunity such as infection, vasculitis, or connective tissue disorder. We could not determine an etiology of encephalitis for two cases. Twenty-five cases fulfilled criteria for pAE, which were classified using the Graus diagnostic algorithm for AE (4): 9 cases diagnosed with definite NMDARE, 14 cases diagnosed with other AE, and 2 cases classified as “reconsider diagnosis.” Two out of the 14 cases with other AE were excluded from the following EEG analysis because of insufficient EEG data. Eventually, we analyzed EEGs from 21 cases, including 9 cases with NMDARE and 12 cases with other AEs. ADEM, acute disseminated encephalomyelitis; AE, autoimmune encephalitis; BBE, Bickerstaff’s brainstem encephalitis; EEG, electroencephalogram; HE, Hashimoto’s encephalopathy; LE, limbic encephalitis; NMDARE, anti-N-methyl-d-aspartate receptor encephalitis; pAE, possible autoimmune encephalitis.

The study is a retrospective case–control study and was approved by the ethics committee of the Nihon University Itabashi Hospital. The details of patients’ selection and classification are depicted in **Figure 1**. Briefly, we reviewed the clinical records of 90 patients with acute encephalitis who were treated in our hospital between January 2014 and October 2020. Then, we implemented in-house antibody screening with patients’ cerebrospinal fluid (CSF), which was followed by confirmatory tests for onconeural and neuronal surface antibodies (**Supplementary Methods**). We enrolled the patients who fulfilled the diagnostic criteria for possible AE (pAE) as defined by Graus et al. (pAE criteria) (4) and extracted 25 patients who fulfilled the pAE criteria. Then, 23 patients with fully accessible clinical records were enrolled. We classified the pAE patients into 9 definite NMDARE and 12 other AEs, which included definite autoimmune limbic encephalitis (LE), definite AEDM, definite AE, definite Bickerstaff’s encephalitis (BBE), Hashimoto’s encephalopathies (HE), and antibody negative probable AE (4). Two patients were eventually unclassified into any group of AEs, namely, concluded as “reconsider diagnosis”.

### Assessment of Clinical Features

The clinical features that included demographics, main syndrome, and complementary data that include findings of CSF tests, antibody tests for antineuronal antigens, cranial MRI, EEG, treatments, and outcomes were compared between the groups of NMDARE and other AEs.

### EEG Setting, Data Acquisition, and Analyses

EEG was initially recorded upon admission with a multichannel EEG machine (Nihon Kohden Corporation, Tokyo, Japan) obtained by certified technologists. Details on EEG settings and qEEG analyses are summarized in **Supplementary Methods** and **Supplementary Figure 1**.

Briefly, all clinical EEG recordings were conducted using 0.5 Hz low- and 60 Hz high-frequency filters. The EEG PV analyses of qEEGs were implemented using the initial EEG records. PVs for each frequency were calculated *via* fast Fourier transform (FFT) analysis with EMSE^®^ version 5.5 (Cortech Solutions, Inc., NC, USA) software. PVs were classified into the frequency bands as alpha (8.0–13.0 Hz), beta (13.1–30.0 Hz), theta (4.0–7.9 Hz), or delta (0.5–3.9 Hz) band. The PV proportion of each frequency band is shown in **Supplementary Figure 2**. With the comparative analyses of PV, a novel qEEG parameter called the fast slow ratio (FSR), which was defined as PV of beta band/PV of theta and delta bands, was established by comparing PVs. FSR was compared between the groups.

We also explored the influence of sedative drugs, such as consistent midazolam and propofol infusion, on qEEG findings. We then evaluated the value of FSR between the groups in the patients without both of the sedative drugs.

### Analyses of Diagnostic Accuracy for proNMDARE Criteria and FSR

We evaluated how helpful a novel qEEG index FSR is to distinguish NMDARE from other AEs when compared to the criteria of “probable NMDARE” described by Graus (proNMDARE criteria) (4). The proNMDARE criteria were rapid onset of at least four of six major groups of symptoms: (1) abnormal behavior or cognitive dysfunction, (2) speech dysfunction, (3) seizures, (4) movement disorders, (5) decreased level of consciousness, and (6) autonomic dysfunction or central hypoventilation, associated with either abnormal EEG findings, CSF pleocytosis, or oligoclonal bands. Specificity and sensitivity of diagnosis were calculated when either FSR or proNMDARE criteria were applied to 9 NMDARE and 12 other AEs patients.

### Statistical Analysis

Mann–Whitney *U* test and Fisher’s exact test were used to assess statistical significance in the different clinical features for non-normally distributed continuous data and categorical data, respectively. Mann–Whitney *U* test was also used to compare FSR values between groups. Receiver operating characteristic (ROC) curve analyses were implemented to determine specificity and sensitivity of an appropriate threshold value in discriminating NMDARE from other AEs. A threshold *p*-value of 0.05 indicated statistical significance in all cases.

## Results

This study included 21 patients with AE, whose clinical records and complementary tests including EEG could be fully accessed. The patients were classified into 9 with NMDARE and 12 with other AEs, who were also classified into six categories of AE according to Graus criteria (4) (**Figure 1**).

### Comparison of the Clinical Features of Patients With NMDARE and Other AEs

**Table 1** shows a summary of demographics, main symptoms, complementary tests, treatments, and outcomes of the patients with NMDARE (*n* = 9) and other AEs (*n* = 12); detailed clinical courses of seven representative cases can be found in **Supplementary Results**. Demographic data revealed that all but one NMDARE were female, while six with other AEs were female. The median age was 21 (16–50) years and 37.5 (17–53) years. Prodrome emerged in seven and nine patients with NMDARE and other AEs, respectively. Speech dysfunction (9/9 vs. 4/12, *p* = 0.005) and movement disorders (6/9 vs. 1/12, *p* = 0.016) were significantly more frequent in the patients with NMDARE than in those with other AEs. The frequencies of other symptoms that included abnormal behavior or cognitive dysfunction, decreased level of consciousness, seizures, and autonomic dysfunction/central hypoventilation were not significantly different between the groups.

**Table 1:** Comparison of the clinical features between NMDARE and other AEs.

	NMDARE (*n* = 9)	Other AEs (*n* = 12)	*p*-value
Sex, female	8	6	0.159
Age, years, median (range)	21 (16–50)	38 (17–71)	0.056
Hospitalization, day, median (range)	74 (37–210)	44 (19–197)	0.164
Follow up period, months, median (range)	23 (8–81)	14.5 (4–64)	0.474
Symptoms			
Prodrome	7	9	1.000
Abnormal behaviour or cognitive dysfunction	9	11	1.000
Speech dysfunction	9	4	0.005**
Seizures	6	4	0.198
Movement disorder, dyskinesias, or rigidity/abnormal postures	6	1	0.016*
Decreased level of consciousness	6	10	0.610
Autonomic dysfunction or central hypoventilation	4	9	0.203
CSF with pleocytosis (cell >5/μl)	8	9	0.603
MRI abnormality	2	9	0.030*
EEG			
Range from onset, day, median (range)	8 (2–23)	11.5 (1–32)	0.452
EEG findings			
Focal/diffuse slowing	9	12	1.000
Beta activity^1^	5	1	0.046*
Epileptiform activity	1	1	1.000
Extreme Delta Brush	1	0	0.429
Rhythmic Delta Activity^2^	3	7	0.387
Lateralized Periodic Discharge	0	1	1.000
Intractable epilepsy (AEDs≧3)	3	1	0.272
Sedative drug required	4	5	1.000
Immunotherapies			
IVMP	9	12	1.000
IVIg	8	6	0.159
Plasma exchange	1	1	1.000
Second line immunotherapies	5	0	0.006**
Modified Rankin Scale			
Peak (range)	5 (1–5)	5 (2–5)	0.603
Current (range)	3 (0–4)	3 (0–4)	0.555

NMDARE, anti-N-methyl-d-aspartate receptor encephalitis; AEs, autoimmune encephalitis; CSF, cerebrospinal fluid; MRI, magnetic resonance imaging; AEDs, antiepileptic drugs; IVMP, intravenous methylprednisolone; IVIg, intravenous immunoglobulins; mRS, modified Rankin scale.

^1^Beta activity included diffuse or focal beta activity and excessive beta activity.

^2^RDA included focal or generalized and intermittent or continuous RDA. *p < 0.05, ** p < 0.01.

**Figure 2:**
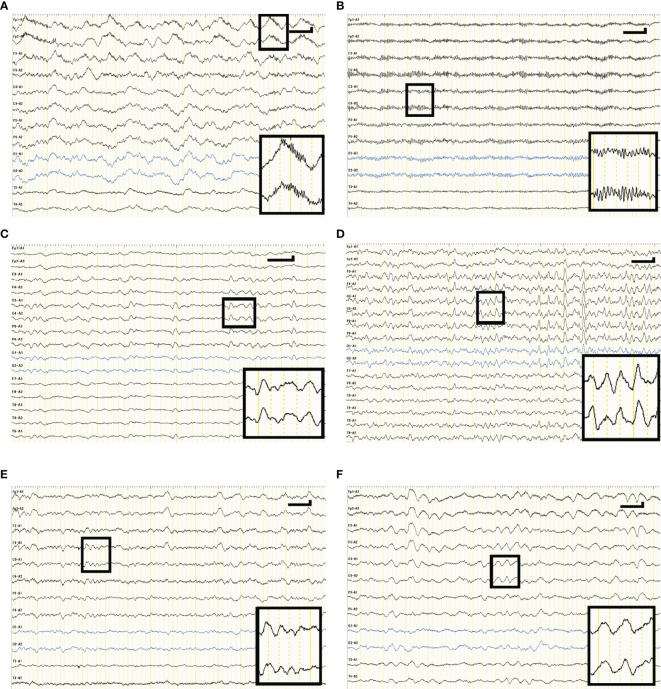
Representative EEG waveforms of cases with NMDARE **(A, B)**, definite autoimmune encephalitis **(C, D)**, ADEM **(E)**, and LE **(F)**. **(A)** shows extreme delta brush consisting of rhythmic beta activity upon rhythmic delta activity—a waveform specific to patients with severe NMDARE—observed in case 1 in NMDARE group. **(B)** shows excessive beta activity observed in case 6 in the NMDARE group. **(C)** shows background slowing and intermittent rhythmic delta activity observed in case 5 of the other AEs group. **(D)** shows background slowing and generalized rhythmic delta activity observed in case 6 of the other AEs group. **(E)** shows background slowing whose frequency was 3–5 Hz observed in case 7 of the other AEs group. **(F)** shows frontal intermittent rhythmic delta activity observed in case 9 of the other AEs group. Vertical and horizontal bars in each panel indicate 50 µV and 1 s, respectively. ADEM, acute disseminated encephalomyelitis; LE, limbic encephalitis.

Complementary tests detected CSF pleocytosis in 8 and 10 patients, respectively, in the NMDARE and other AEs groups. EEGs were recorded at 8 (2–23) days and 12 (1–32) days in the NMDARE and other AEs groups, respectively; representative EEG findings from each group are shown in **Figure 2**. Focal/diffuse slow activity was observed in all 21 patients. Diffuse beta activity occurred more frequently in the NMDARE group than in other AEs (5/9 vs. 1/12, *p*= 0.046). EDB was observed in one patient with NMDARE but in no patients with the other AEs. One patient with other AEs showed periodic lateralized epileptiform discharges, though the frequency of rhythmic delta activity was similar between the groups. Cranial MRI showed specific lesions in two patients with NMDARE, and MRI-specific lesions were more frequent in the other AEs group (2/9 vs. 9/12, *p* = 0.030), which included demyelinating lesions in ADEM and limbic lesions in autoimmune LE.

All patients were treated with the first-line immunotherapies that included intravenous methyl prednisolone pulse, intravenous immunoglobulins, and plasmapheresis. Five with NMDARE were resistant to first-line immunotherapies, and all were treated with several cycles of intravenous cyclophosphamide pulse therapies. One-third of NMDARE patients and one out of twelve patients with other AEs had intractable epilepsy. Four and five patients, respectively, received sedative drugs to control the confused non-reassuring condition.

Median hospitalization period was 74 (37–210) and 44 (19–197) days in NMDARE and for other AEs, respectively (*p* = 0.164). Outcomes evaluated with modified Rankin scale (mRS) in the peak and current status were not significantly different between the groups.

### Novel qEEG Parameter FSR and ROC Curve Analyses

**Figure 3:**
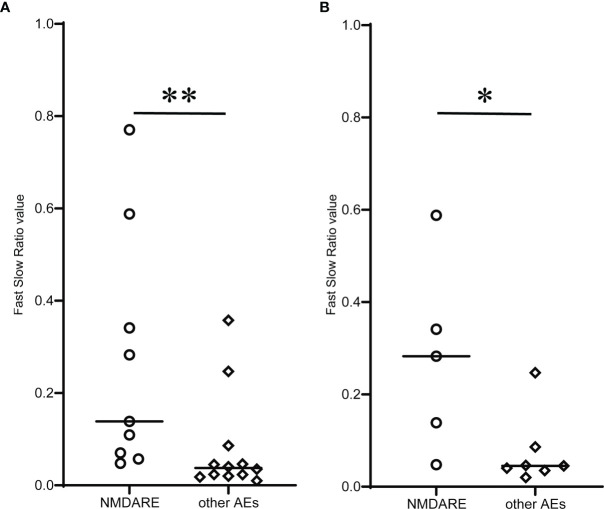
Comparison of novel qEEG parameter Fast Slow Ratio (FSR) between NMDARE and other AEs groups. **(A)** shows FSR of all patients, and **(B)** shows FSR of sedative-free population in each group. Circles and rhombuses indicate FSR of individual cases of NMDARE and other AEs groups, respectively, and horizontal bars indicate the median of each group. Significantly higher FSR in the NMDARE group than other AEs group was observed both when all patients were included and when only the sedative-free population was included. The statistical significance was tested using Mann–Whitney *U* test. *p < 0.05, **p < 0.01.

FSR, or the PV ratio between fast and slow EEG components, was compared across groups (**Figure 3**). The median FSR was significantly higher in the NMDARE group than the other AEs (0.139 vs. 0.029, *p* = 0.004) (**Figure 3A**). The FSR in sedative-free patients was also greater (0.283 vs. 0.040, *p* = 0.018) in NMDARE (*n* = 5) patients than in other AEs (*n* = 7) (**Figure 3B**).

**Figure 4:**
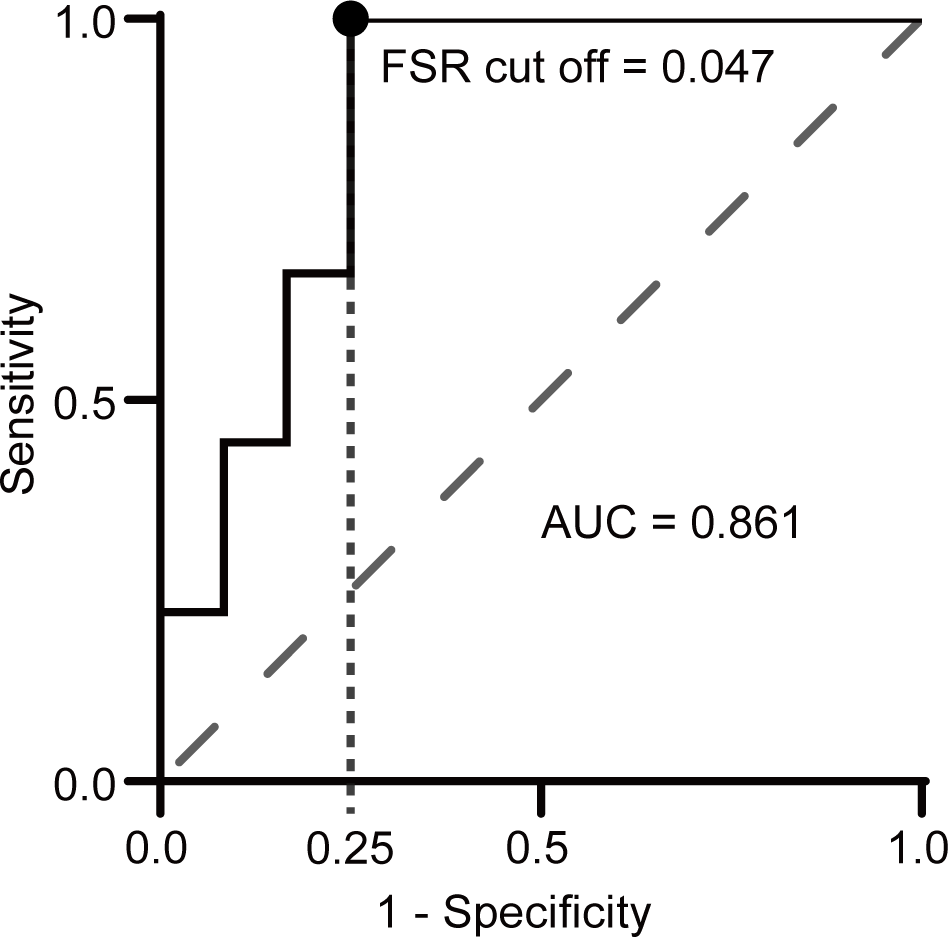
Receiver operating characteristic (ROC) curve analysis of FSR for discriminating NMDARE from other AEs. A circle indicates the point closest to the upper left corner. The AUC was 0.86 (95% CI 0.70–1.00), and the FSR cutoff value of 0.047 yielded a sensitivity of 1.00 and a specificity of 0.75.

We performed ROC curve analysis to distinguish NMDARE from other AEs using FSR, where the ROC curve area was 0.861 (95% CI 0.698–1.000), and the FSR cutoff value of 0.047 yielded a specificity of 0.75 and a sensitivity of 1.00 when indicating NMDARE (**Figure 4**).

### Comparative Analyses of Well-Characterized Clinical Indicator and FSR for the Distinction of NMDARE From Other AEs

We evaluated the diagnostic usefulness of the novel qEEG index FSR compared with proNMDARE criteria (4). Results of qEEG analyses for all 21 individuals are shown in **Supplementary Table 1**. Comparative analyses of the proNMDARE criteria and the FSR are shown in **Table 2**. Two-thirds of patients with definite NMDARE while only five of twelve with other AEs fulfilled proNMDARE criteria. The sensitivity and specificity for the diagnosis of NMDARE according to proNMDARE criteria were 0.67 (6/9) and 0.58 (7/12), respectively. Comparatively, all nine patients with definite NMDARE had higher FSR values than the cutoff of 0.047—this was the case for only three of twelve patients with other AEs. The sensitivity and specificity for the diagnosis of NMDARE using the FSR cutoff value are 1.00 (9/9) and 0.75 (3/12), respectively. Thus, the positive likelihood ratio for the diagnosis of NMDAR with the FSR above cutoff was greater than that of proNMDARE criteria (4.00 vs. 1.60). In addition, the positive predictive value for the diagnosis of NMDARE with proNMDARE criteria and FSR above cutoff is 0.55 (6/11), and 0.75 (9/12), while negative predictive value was 0.70 (7/10) and 1.00 (9/9), respectively.

**Table 2:** Number and frequency of patients who met criteria of probable NMDARE (proNMDARE) and patients whose FSR was higher than our cutoff value.

		Higher FSR than cutoff		
		Yes, *n* (%)	No, *n* (%)	Total, *n* (%)
NMDARE group (*n* = 9)				
proNMDARE	Yes, *n* (%)	6 (67)	0 (0)	6 (67)
	No, *n* (%)	3 (33)	0 (0)	3 (33)
	Total, *n* (%)	9 (100)	0 (0)	9 (100)
other AEs group (*n* = 12)				
proNMDARE	Yes, *n* (%)	1 (8)	4 (33)	5 (42)
	No, *n* (%)	2 (17)	5 (42)	7 (58)
	Total, *n* (%)	3 (25)	9 (75)	12 (100)

## Discussion

We reviewed the clinical records of 90 patients who fulfilled the diagnostic criteria for encephalitis and encephalopathy (9) and extracted 25 patients who met the pAE criteria. Twenty-three were classified into 9 patients with NMDARE and 12 patients with other AEs according to the criteria (4); two patients were eventually excluded for classifying as “reconsider diagnosis”. The clinical features of all 21 patients diagnosed with AE were evaluated, and initial qEEG indices were compared between the NMDARE and other AEs groups. Our study revealed significantly more frequent speech dysfunction and movement disorders among the NMDARE patients. A novel qEEG index—FSR, which was defined as the PV ratio of beta and slow frequency bands—distinguished the NMDARE from other AEs with reasonable specificity and sensitivity.

Antibodies that flock to neuronal surface antigens trigger both paraneoplastic and non-paraneoplastic AE, which includes a variety of inflammatory brain disorders (2), accounting for 21%–39% of acute encephalitis (10–12). Since Graus et al. (4) developed an algorithm for the diagnosis of AE, which consisted of syndrome-based approach and antibody testing, several studies have been reported that classified the encephalitis cohort into specific conditions of autoimmune etiology by the criteria (5, 12–14). Given that the AE defined by the criteria is not a single disease entity, it is no wonder the proportion of each specific condition is varied among the studies. For instance, the proportion of NMDARE accounted for 17%–67% of AE and was on average 48% (43/90) across three studies (5, 12, 13). Our study agrees with others in that the proportion of AE encephalitis was 26% (23/90), of which NMDARE accounted for 39% (9/23) of AE. Recent studies also recommend diagnosing AE by immunolabeling with the rat brain tissue (tissue-based assay: TBA) and/or culturing live primary neurons to screen a series of neuronal surface antibodies (NSAs) in patients’ CSF and serum (15, 16). Accordingly, we analyzed all 90 paired samples (both CSF and serum) by using in-house screening assays; 11 positive patients, whose samples produced neuropil immunostaining on TBA and detected immunofluorolabeled neurons on Live-neuron assay, were then classified into nine NMDARE, of whom two (cases 5 and 6 in other AEs) screened positives without detection of the 7 types of commercially available antigens on the cell-based assay (**Supplementary Table 1**).

Previous studies reported that speech dysfunction and movement disorders were more frequent in NMDARE than other AEs (17, 18) (**Table 1**). Consistently, we also identified speech dysfunction (100% vs. 33%, *p* = 0.005) and movement disorders (67% vs. 8%, *p* = 0.016) as the characteristic symptoms of NMDARE when compared to other AEs, though the cohort size was relatively small. We found highly frequent CSF pleocytosis in NMDARE cases (89%), which agrees with a previous large cohort study (3), but found no significant difference between the groups. We also found that the specific abnormality on cranial MRI was less frequent in patients with NMDARE than that on patients with other AEs (22% vs. 75%).

We analyzed qEEGs by comparing PVs in each frequency band between groups; this method was theoretically established for diagnosing other neuropsychiatric disorders (19–22). The findings in EEGs from AE patients have found a fast component (beta activity) in 25%–50% of those with NMDARE (7, 23–25) but not other AEs (26, 27). Actually, the present study revealed that diffuse beta activity occurred more frequently in initial EEGs from NMDARE patients (5 vs. 1 patient, *p* = 0.046) than those with other AEs. On the other hand, a recent study more commonly detected a slow component, such as delta activity, in patients with AEs (28–33): 51% in total AEs, 56% in NMDARE, and 40% in other AEs (33). In addition, GRDA with fast activity is more common in NMDARE than in other AEs (34). These findings suggest that comparing the power ratios of fast and slow components can extract NMDARE from patients with AE.

Foff et al. (19) focused on beta and delta activity (beta/delta power ratio: BDPR) in the qEEGs from patients with NMDARE. Their EEG PV analyses distinguished NMDARE from other neurological disorders (specificity 0.60, sensitivity 0.71), although they excluded the AE from the non-NMDARE control group. Meanwhile, the present study exactly focused on definite NMDARE with other AEs according to Graus criteria (4), where FSR distinguished NMDARE from other AEs (FSR: cutoff value 0.047, specificity 0.75, sensitivity 1.00), even in patients who were not administered sedative drugs. These results suggest that FSR derived from qEEG is a promising diagnostic marker when combined with specific syndrome criteria.

This study sought not to clarify the neurophysiological features of FSR but rather to show how the FSR can be used to diagnose NMDARE. The sensitivity of the proNMDARE criteria (4) was 0.67 in our cohort, as three of nine patients with NMDARE were false negatives. This value was consistent with that of other cohort studies (approximately 0.70) (5, 12–14). However, the method using an FSR cutoff value salvaged the three patients who did not meet proNMDARE criteria, thereby achieving a sensitivity of 1.00 (**Table 2**). Focusing on patients who did not meet the proNMDARE criteria (*n* = 10), the pretest probability from NMDAR antibody test was only 0.30 (3/10). When we further focused on patients with higher FSR than the cutoff (*n* = 5), the pretest probability increased to 0.60 (3/5). These results suggest that the diagnostic approach for NMDARE using FSR adding to proNMDARE criteria can contribute to prevent the undervaluation of the candidates who require the antibody tests.

This study also explored the early distinction of NMDARE patients from those who only meet the pAE criteria, which only require the syndrome, cranial MRI, CSF study, and EEG (4). Thus, the pAE criteria can include the patients eventually classified as “reconsider diagnosis,” as was the case for two patients in the present study. We also analyzed how FSR contributed to early distinction of NMDARE from the patients who only fulfilled the pAE criteria despite the small cohort size (*n* = 23, 9 NMDARE vs. 14 other pAEs) (**Figure 1**). The FSR value of NMDARE patients was significantly higher than that of other pAEs in both all-inclusive and sedative-free groups (**Supplementary Figure 3**), and ROC analyses of proNMDARE and FSR revealed that using the FSR cutoff value was both specific and sensitive (0.72 and 1.00, respectively) (**Supplementary Table 2**). Indeed, FSR is a promising qEEG marker for distinguishing NMDARE from the wider range of AE in early stages of disease. Yet, further investigations with larger pAE cohorts are required to confirm its usefulness.

Regarding the EEG findings of NMDARE in the recovery phase, Raja et al. reported that EEG abnormalities remained in 75% of the patients 8 months after onset, although some patients’ EEG findings had returned to normal 1 year after onset (35). In our study, follow-up EEG recordings in the recovery phase were available in 14 patients (7 with NMDARE and 7 with other AEs), and the median period from onset was 29 (range 12–58) and 10 (range 3–65) months in those with NMDARE and other AEs, respectively (*p* = 0.434) (**Supplementary Table 1**). We additionally implemented comparative PV analyses with qEEG in the recovery phase (described in **Supplementary Methods and Results**). Notably, all 14 patients had an increase in the proportion of PV in the alpha band but a decrease in the delta band (**Supplementary Figure 4A**). The individual FSR value in the recovery phase was higher than that in the acute phase (**Supplementary Figures 4B, C**), and the median FSR value did not differ between the NMDARE and other AEs groups (0.270 vs. 0.355, *p* = 0.805). These additional analyses revealed that the FSR derived from qEEG in the recovery phase does not seem suitable for distinguishing NMDARE from other cases of autoimmune encephalitis.

The present study had some limitations, as it was retrospective and had a relatively small cohort of AEs (*n* = 21). No patients with specific NSAs other than NMDAR antibodies (e.g., antibodies against leucine-rich glioma-inactivated 1, contactin-associated protein-like 2, and dipeptidyl-peptidase-like protein 6) were included, though two screening tests of different techniques were used for all patients’ CSF and serum. Moreover, the cohort size classified into other AEs (*n* = 21) was too small to establish the characteristics of the syndromes and complementary results that included qEEG analyses in each autoimmune condition.

## Conclusions

Comparisons between NMDARE and other AEs revealed that the speech dysfunction and movement disorders were more prominent in the NMDARE group. A novel qEEG indicator, FSR, which was defined as the PV ratio of beta and slow frequency bands, distinguished the NMDARE patients from other AEs with a reasonable specificity and sensitivity despite the small cohort size. The FSR derived from qEEG analyses combined with the proNMDARE criteria is a promising early diagnostic marker in patients with NMDAR but should be confirmed in a larger cohort study.

## Data Availability Statement

The original contributions presented in the study are included in the article/**Supplementary Material**. Further inquiries can be directed to the corresponding author.

## Ethics Statement

The studies involving human participants were reviewed and approved by Nihon University Itabashi Hospital, Clinical Research Judging Committee. Written informed consent to participate in this study was provided by the participants’ legal guardian/next of kin. The animal study was reviewed and approved by Nihon University Animal Care and Use Committee.

## Author Contributions

The study was designed by TM and MH. Data were collected by TM, MH, and SH. Data were analyzed by TM. The manuscript was mainly drafted by TM, and SH provided assistance to this work. The manuscript was revised by MH and HN. The study was supervised by MH and HN. All authors contributed to the article and approved the submitted version.

## Funding

This work was supported in part by MHLW Grant Number 19HA1002 and JSPS KAKENHI Grant Number JP20K07875 (MH).

## Conflict of Interest

The authors declare that the research was conducted in the absence of any commercial or financial relationships that could be construed as a potential conflict of interest.

## Publisher’s Note

All claims expressed in this article are solely those of the authors and do not necessarily represent those of their affiliated organizations, or those of the publisher, the editors and the reviewers. Any product that may be evaluated in this article, or claim that may be made by its manufacturer, is not guaranteed or endorsed by the publisher.
