# Supplementary material for: Novel qEEG Biomarker to Distinguish Anti-NMDAR Encephalitis From Other Types of Autoimmune Encephalitis
Source: Front Immunol. 2022 Feb 15;13:845272. doi: 10.3389/fimmu.2022.845272 (PMC8885512; doi:10.3389/fimmu.2022.845272)
Supplement: Supplementary file 1 [file DataSheet_1.pdf]

Supplementary Material

1 Supplementary methods

1.1 In-house assays for screening of NSAs and onconeural antibodies

A series of NSAs (e.g., antibodies against *N*-methyl-d-aspartate receptor [NMDAR], leucine-rich glioma-inactivated 1 [LGI1], contactin-associated protein-like 2 [Caspr2], dipeptidyl-peptidase-like protein 6 [DPPX], and immunoglobulin-like cell adhesion molecule 5 [IgLON5]), and onconeural antibodies (e.g., ANNA1, Yo, Ri, Ma, and CV2) for all 90 patients' CSF and serum samples were screened using the following two techniques: tissue-based assay (TBA) with rat brain sections and immunocytochemistry with rat primary cultured neurons (Live-neuron assay).

1.1.1 In-house TBA

TBA, which involved immunohistochemical analyses of rat brain tissue, was implemented as reported (1). Briefly, adult female Wistar rats were sacrificed without perfusion, and the brain was removed and fixed in 4% paraformaldehyde for 1 h at 4°C, cryoprotected in 40% sucrose for 48 h, embedded in freezing compound media, and snap frozen in isopentane chilled with liquid nitrogen. Thereafter, 6-µm-thick tissue sections were sequentially incubated with 0.3% H_2O_2 for 15 min, 5% goat serum for 1 h, and patients and control CSF (1:2) or serum (1:200) at 4°C overnight. After incubating with biotinylated secondary antibodies against human IgG (1:2000, BA-3000, Vector), the reactivity was developed using the avidin-biotin-peroxidase method. The results of the assay were independently evaluated by two experts (MH and HN) familiar with the immunohistochemical technique, who then classified the samples into "positive (neuropil pattern, astrocytic pattern, white matter pattern, and intracellular pattern)," "negative," or "dubious." The samples categorized into "dubious" required retesting to determine the final TBA results. The samples deemed "positive" were subsequently examined with the confirmation tests described below to determine the specific neuronal antigens.

1.1.2 In-house Live-neuron assay

Rat hippocampal neuronal cultures were prepared as reported (1). Briefly, matured live neurons grown on coverslips were incubated for 1 h at 4°C with patient or control CSF (1:2) or serum (1:80). After removing the media and extensive washing with PBS, neurons were fixed with 4% paraformaldehyde and immunolabeled with Alexa Fluor® 488 goat anti-human IgG (1:1000, A11013, Invitrogen). The results were photographed using a fluorescent microscope (BZ-X810, KEYENCE, Osaka, Japan). The results of the assay were evaluated by an expert (MH) familiar with the indirect immunofluorescence assay, who then classified the samples into "positive" or "negative." The samples classified as "positive" were subsequently examined with the confirmation tests below to determine the specific neuronal surface antigens.

1.2 Confirmation tests of NSAs and onconeural antibodies with commercially available tests

For patients with a positive result during in-house TBA and/or Live-neuron assay, subsequent confirmation tests using commercially available cell-based assay (CBA) for 7 neuronal surface antigens (NMDAR, α-amino-3-hydroxy-5-methyl-4-isoxazolepropionic acid receptor, LGI1, Caspr2,

gamma-aminobutyric acid receptor type B, DPPX, and IgLON5) (BIOCHIP, Euroimmun, performed by Labor Berlin) and/or commercially available line blot assays for 12 onconeural antigens (EUROLINE, Euroimmun, Lübeck, Germany) were performed.

1.3 Detection of other types of autoantibodies associated with autoimmune encephalitis

Antibodies against aquaporin-4 and myelin oligodendrocyte glycoprotein in the serum were screened using CBA (Cosmic Corporation Co., Ltd., Tokyo, Japan) for all 25 patients who fulfilled the diagnostic criteria for possible AE (2). Similarly, antibodies against thyroid peroxidase, thyroglobulin, and GQ1b were tested for the serum samples of the 25 patients.

1.4 EEG power value analysis

EEG power value (PV) analyses, which have been employed for the purpose of evaluating various neuropsychiatric disorders (3-5), were conducted using the following procedure (Supplementary Figure 2). First, after EEG recording on admission, we randomly selected 10 regions of 13-second artifact-free/seizure-free areas (3, 6). We then calculated the PVs for each frequency for each of the 10 selected regions from channels C3-C4 via fast Fourier transform (FFT) with the nonoverlapping Hanning bins, using EMSE® version 5.5 (Cortech Solutions, Inc., NC, USA) software. The PVs at each frequency were summed up across the 10 selected regions, and then summed up according to four frequency bands of alpha (8.0–13.0 Hz), beta (13.1–30.0 Hz), theta (4.0–7.9 Hz), or delta (0.5–3.9 Hz) bands. The calculated PVs of the three frequency bands (beta, theta, delta) (Supplementary Figure 2) were used to produce our novel qEEG parameter, which we termed the "Fast Slow Ratio" (FSR; PV of beta band/PV of theta and delta bands) for each patient. Finally, we compared the FSR values between the NMDARE and other AEs groups, and further conducted receiver operating characteristic (ROC) curve analysis for discriminating NMDARE from other AEs.

1.5 Comparative PV analyses between NMDARE and other AEs in acute and recovery phase

We comparatively analyzed the EEG power value (PV) in the NMDARE and other AEs groups between the acute and recovery phases. On EEG recording, the acute phase was defined as that initially recorded upon admission, whereas the recovery phase depended on the patients' status 3 months or more after onset. PV analyses were implemented using the aforementioned procedures. Given that most of the patients in the recovery phase could follow instructions involving eye opening and closing, we extracted the 10 selected regions recorded during the eyes-closed resting-state conditions. The FSR values were compared between the NMDARE and other AEs group in the acute and recovery phase, and statistical differences between the groups were tested using the Mann–Whitney U test, with a threshold p value of 0.05 indicating statistical significance.

2 Supplementary Results

2.1 Clinical course of the representative cases

Here, we present the detailed clinical courses of seven representative cases: one from the anti-*N*-methyl-d-aspartate receptor encephalitis (NMDARE) group and six from the other autoimmune encephalitis (other AEs) group.

2.1.1 Example 1 (case 1 in NMDARE group in Supplementary Table 1)

A 24-year-old woman developed symptoms of NMDARE that started with cognitive dysfunction and psychosis, followed by speech disorder, seizures, involuntary movements, decreased level of consciousness, and hypoventilation. The CSF test indicated pleocytosis. The tissue-based assays (TBAs), including indirect immunolabeling with rat frozen brain sections and live primary hippocampal neurons1, revealed a positive result, and anti-NMDAR antibody was detected in the CSF using cell-based assays. Cranial MRI revealed non-specific lesions. The initial EEG showed background slowing, intermittent generalized beta activity, and extreme delta brush (EDB) (Figure 2A). Consequently, she fulfilled the diagnostic criteria for definite NMDARE (2). During 210 days of hospitalization, she required sedative drugs, three or more antiepileptic drugs, and mechanical ventilation. She received intravenous corticosteroids, plasma exchange, and immunoglobulins as first-line immunotherapies, and further received multiple cycles of intravenous cyclophosphamide as a second-line immunotherapy. Her poorest mRS status was 5, which improved to 3 at the time of discharge. No relapse occurred and her seizures were well controlled during 81 months of follow-up after discharge.

2.1.2 Example 2 (case 6 in other AEs group in Supplementary Table 1)

An 18-year-old woman developed symptoms of AE that started with pyrexia, headache, and cognitive dysfunction, followed by speech dysfunction, decreased level of consciousness, seizure, and hypoventilation. The CSF test indicated pleocytosis. TBAs revealed a positive result in the CSF. Cranial MRI revealed no lesions. The initial EEG showed background slowing and generalized rhythmic delta activity (Figure 2D). Consequently, she fulfilled the diagnostic criteria for definite AE (2). During 33 days of hospitalization, she required more of three antiepileptic drugs and mechanical ventilation. She received intravenous corticosteroids as first-line immunotherapies. Her poorest mRS status was 5, which improved to 2 at the time of discharge. No relapse occurred during 25 months of follow-up after discharge.

2.1.3 Example 3 (case 7 in other AEs group in Supplementary Table 1)

A 36-year-old woman developed symptoms of acute disseminated encephalomyelitis (ADEM) that started with pyrexia, headache, dysfunction of the bladder and bowel, and cognitive dysfunction, followed by a decreased level of consciousness. The CSF test indicated pleocytosis. TBAs revealed a negative result in the CSF. Cranial MRI revealed hyperintensity of the bilateral thalami and basal ganglia in the T2-weighted image. The initial EEG showed background slowing and frontal intermittent rhythmic delta activity (FIRDA) (Figure 2E). Consequently, she fulfilled the diagnostic criteria for definite ADEM (2). During 33 days of hospitalization, she required mechanical ventilation and received intravenous corticosteroids as first-line immunotherapies. Her poorest mRS status was 4, which improved to 1 at the time of discharge. No relapse occurred during 12 months of follow-up after discharge.

2.1.4 Example 4 (case 9 in other AEs group in Supplementary Table 1)

A 46-year-old woman developed symptoms of limbic encephalitis (LE) that started with pyrexia, abnormal behavior, and cognitive dysfunction, followed by speech dysfunction, decreased level of consciousness, hypoventilation, and urinary retention. The CSF test indicated pleocytosis. TBAs revealed negative results in both the serum and CSF. Cranial MRI revealed hyperintensities in the bilateral temporal regions in the diffusion-weighted image and the T2-weighted image. The initial EEG showed background slowing and FIRDA (Figure 2F). Consequently, she fulfilled the diagnostic criteria for definite acute autoimmune LE (2). During 197 days of hospitalization, she required

sedative drugs and mechanical ventilation, and received intravenous corticosteroids and immunoglobulins as first-line immunotherapies. Her poorest mRS status was 5, which improved to 4 at the time of discharge. She showed no relapse during the 6 months of follow-up after discharge, but required assistance in daily life because of severe sequelae.

2.1.5 Example 5 (case 3 in other AEs group in Supplementary Table 1)

A 31-year-old man developed symptoms of Bickerstaff's brainstem encephalitis (BBE) that started with cognitive dysfunction, dysarthria followed by bilateral external ophthalmoplegia, muscle weakness, decreased level of consciousness, ataxia, and hypoventilation. The CSF test indicated pleocytosis, and anti-GQ1b antibody was detected in the serum. Cranial MRI revealed no lesions. The initial EEG showed mild background slowing. Consequently, he fulfilled the diagnostic criteria for definite BBE (2). During 37 days of hospitalization, he required mechanical ventilation, and received intravenous corticosteroids and immunoglobulins as first-line immunotherapies. His poorest mRS status was 5, which improved to 2 at the time of discharge. No relapse occurred during 26 months of follow-up after discharge.

2.1.6 Example 6 (case 4 in other AEs group in Supplementary Table 1)

A 49-year-old woman developed symptoms of Hashimoto's encephalopathy (HE) that started with abnormal behavior, pyrexia, hallucinations followed by seizure, myoclonus, and decreased level of consciousness. The CSF test was normal. TBAs revealed a negative result in the CSF. Both anti-thyroid peroxidase antibody and anti-thyroglobulin antibody were detected in the serum, which is associated with mild hyperthyroidism. Cranial MRI revealed non-specific lesions in the white matter. The initial EEG showed background slowing and low-voltage generalized beta activity. Consequently, she fulfilled the diagnostic criteria for HE (2). During 54 days of hospitalization, she required sedative drugs and received intravenous corticosteroids as first-line immunotherapies. Her poorest mRS status was 5, which improved to 3 at the time of discharge. No relapse occurred and seizures were well controlled during 64 months of follow-up after discharge.

2.1.7 Example 7 (case 12 in other AEs group in Supplementary Table 1)

A 45-year-old man developed symptoms of AE that started with pyrexia, followed by decreased level of consciousness and hypoventilation. The CSF test indicated pleocytosis. TBA revealed negative results both in the serum and CSF. Cranial MRI revealed hyperintensity of the left thalamus and pons in the T2-weighted image. The initial EEG showed background slowing and FIRDA. Consequently, he fulfilled the diagnostic criteria for autoantibody-negative but probable autoimmune encephalitis (2). During 42 days of hospitalization, he required sedative drugs and mechanical ventilation, and received intravenous corticosteroids as first-line immunotherapies. His poorest mRS status was 5, which improved to 4 at the time of discharge. No relapse occurred during 4 months of follow-up after discharge.

2.2 Comparative PV analyses between NMDARE and other AEs in acute and recovery phase

Follow-up EEG data in the recovery phase were available for 14 patients (7 with NMDARE and 7 with other AEs). The median period from onset was 29 (range 12–58) and 10 (range 3–65) months in those with NMDARE and other AEs, respectively (p=0.434) (Supplementary Table 1). The proportion of PV in each frequency band is shown in Supplementary Figure 4A. In the recovery phase, all 14 patients showed an increase in the proportion of PV in the alpha band but a decrease in the delta band. The individual FSR value in the recovery phase was higher than that in the acute

phase in both groups (Supplementary Table 1, Supplementary Figure 4B and 4C). The median FSR in the recovery phase did not differ between the NMDARE and other AEs groups (0.270 vs. 0.355, p=0.805).

3 Supplementary Figures

3.1 Supplementary Figure 1. Flowchart of EEG recording and analysis

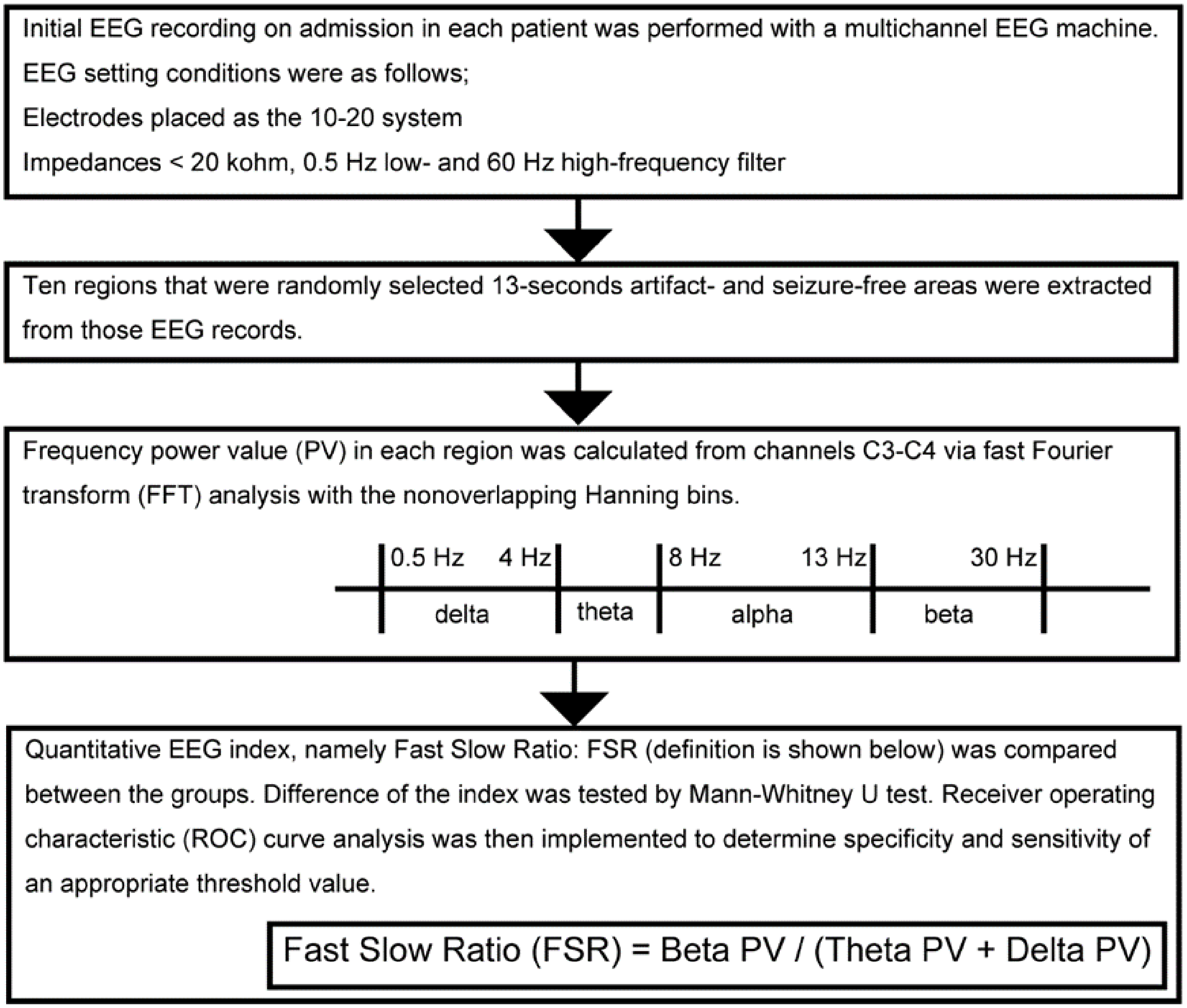

After EEG recording on admission, we randomly selected 10 regions of 13-second artifact-free/seizure-free areas, and conducted fast Fourier transform (FFT) to yield the total power value (PV) for each frequency for each of the 10 selected regions. The PVs were summed up according to the frequency bands such as alpha (8.0–13.0 Hz), beta (13.1–30.0 Hz), theta (4.0–7.9 Hz), or delta (0.5–3.9 Hz) bands. The PVs of each frequency bands were used to product Fast Slow Ratio (FSR) for each patient. We compared the FSR values between NMDARE and other AE groups, and further conducted receiver operating characteristic (ROC) curve analysis for discriminating NMDARE from other AEs.

3.2 Supplementary Figure 2. Power values (PVs) of each frequency bands for each individual case

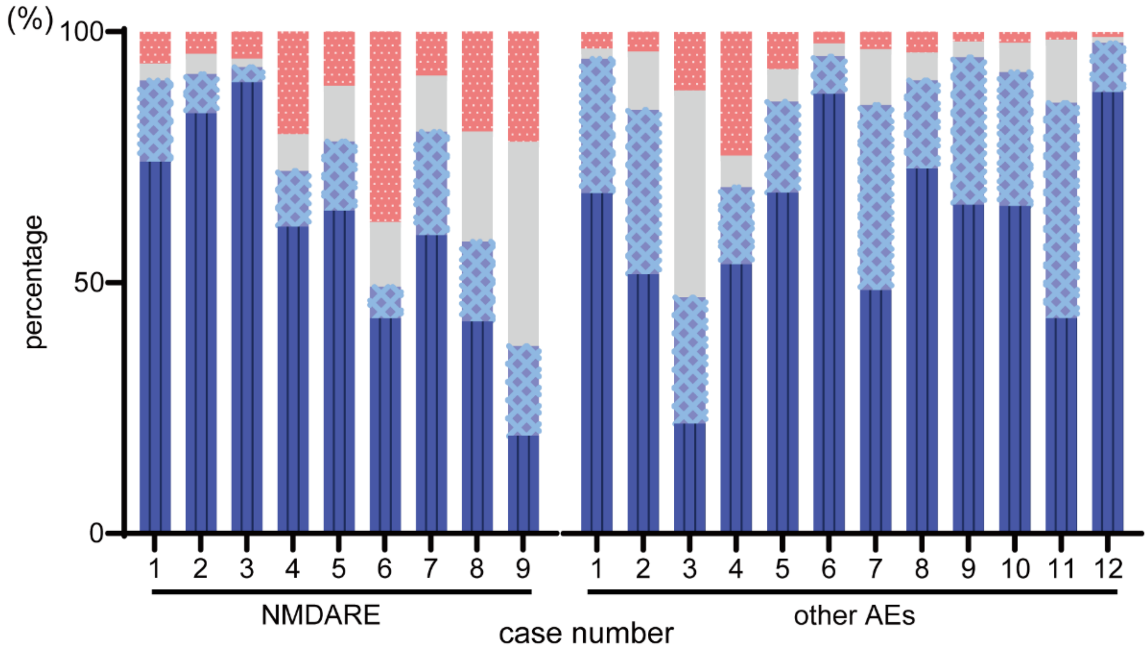

The left 9 bars and the right 12 bars indicate the PV of individual cases in the NMDARE group and other AEs group, respectively. In each bar, the four colors represent the percentage of PV for each frequency band: blue represents the delta band; light blue represents the theta band; light gray represents the alpha band; and red represents the beta band. NMDARE: anti-*N*-methyl-d-aspartate receptor encephalitis, other AEs: other types of autoimmune encephalitis.

3.3 Supplementary Figure 3. Comparison of novel qEEG parameter Fast Slow Ratio (FSR) between NMDARE group and possible autoimmune encephalitis other than NMDARE (other pAEs) group

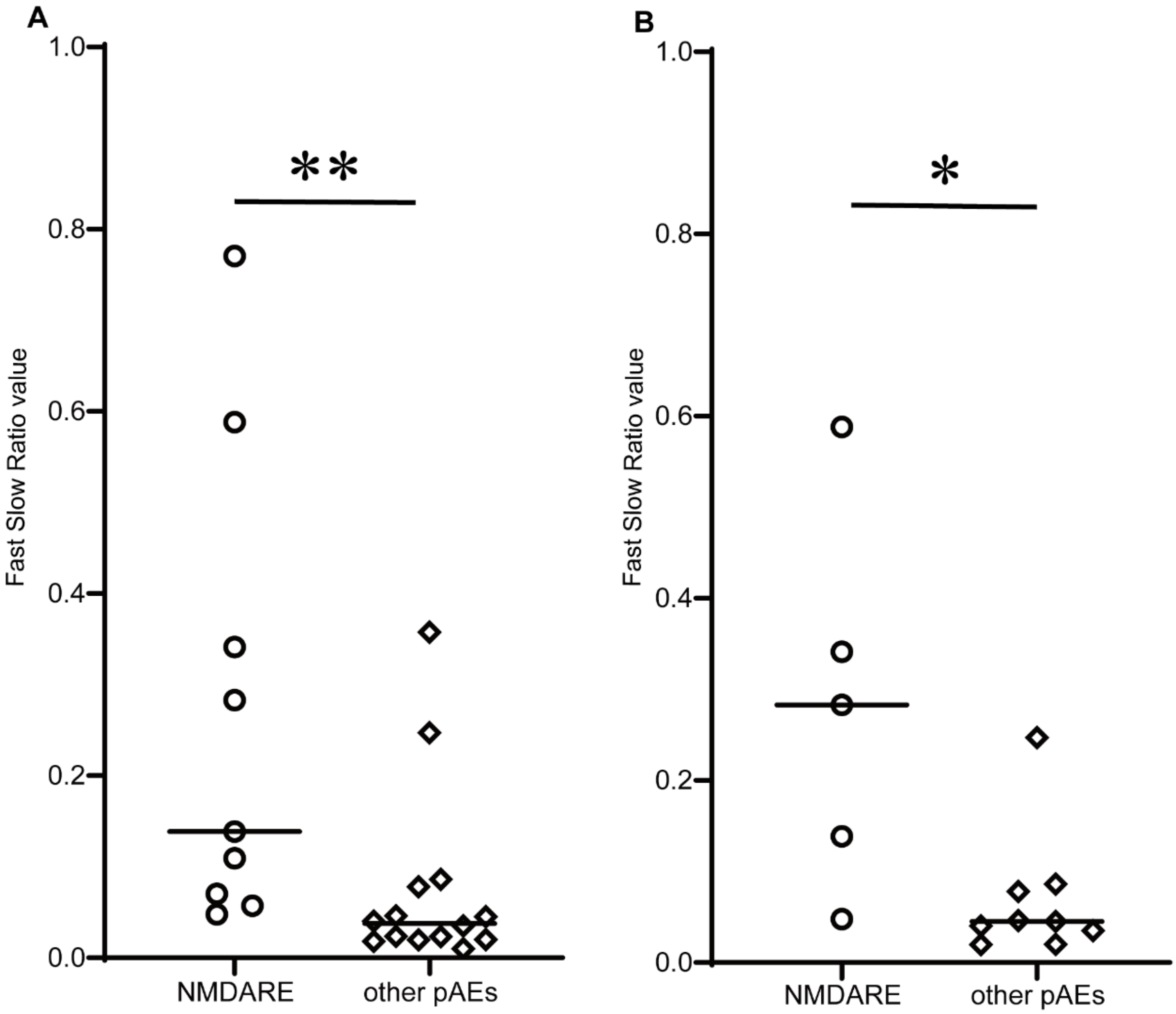

Comparison of novel qEEG parameter Fast Slow Ratio (FSR) between NMDARE group and possible autoimmune encephalitis other than NMDARE (other pAEs) group. NMDARE group contained 9 patients who fulfilled criteria for definite NMDARE, and other pAEs group contained 14 patients who fulfilled criteria for possible AE but not fulfilled criteria for definite NMDARE. Panel A shows FSR of all patients, and panel B shows FSR of sedative free population in each group. Circles and rhombuses indicate FSR of individual cases of NMDARE and other pAEs groups respectively, and horizontal bars indicate median of each group. Significantly higher FSR in NMDARE group than other pAEs group was observed both when all patients were included and when only sedative free population was included. The statistical significance was tested using Mann–Whitney *U* test. $*p < 0.05$, $**p < 0.01$

3.4 Supplementary Figure 4. Power values (PVs) of each frequency bands in the recovery phase, and comparison of FSR between the acute and recovery phases

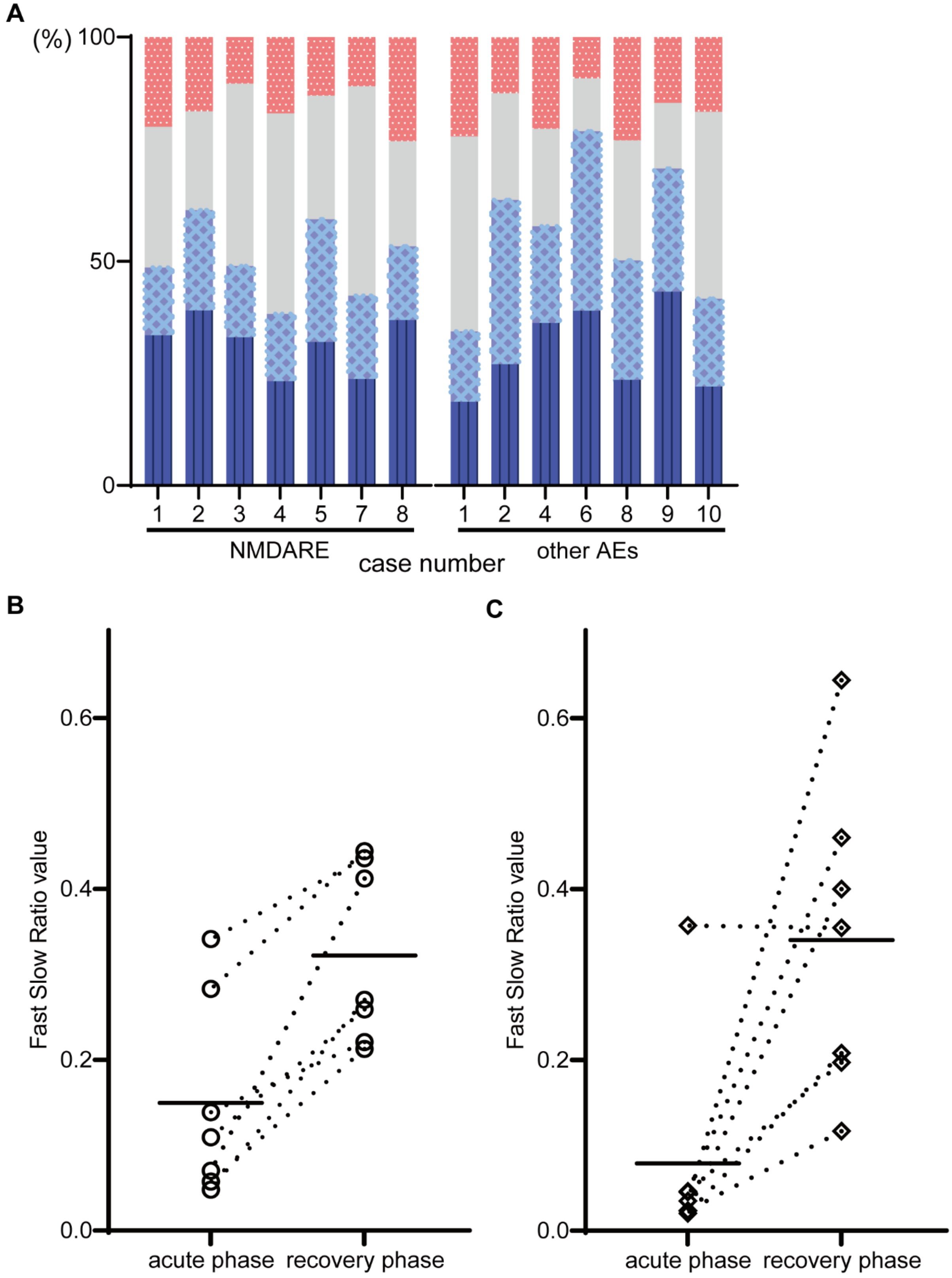

Follow-up EEG in the recovery phase were available for 14 patients (7 with NMDARE and 7 with other AEs). The left 7 bars and the right 7 bars on panel A indicate the PV of individual cases in the recovery phase in the NMDARE and other AEs groups, respectively. In each bar, the four colors represent the percentage of PV for each frequency band: blue represents the delta band; light blue represents the theta band; light gray represents the alpha band; and red represents the beta band. In the recovery phase, all 14 patients showed an increase in the proportion of PV in the alpha band but a decrease in the delta band. Panel B shows the FSR of the NMDARE group, and panel C shows the FSR of the other AEs group. The circles in panel B (NMDARE) or rhombuses in panel C (other AEs) connected by dashed lines represent the FSR in the acute (left) or recovery phase (right), and each horizontal bar indicates the median FSR. The individual FSR value in the recovery phase was higher than that in the acute phase in both groups (4B and 4C). NMDARE: anti-*N*-methyl-d-aspartate receptor encephalitis, other AEs: other types of autoimmune encephalitis, FSR: Fast Slow Ratio.

4 Supplementary Tables

4.1 Supplementary Table 1. Demographic and clinical data of 21 patients with autoimmune encephalitis

case	diagnosis	sex	age (years)	hospitalization (days)	Follow up period (months)
NMDARE					
1	NMDARE	F	24	210	81
2	NMDARE	M	18	129	23
3	NMDARE	F	21	103	20
4	NMDARE	F	19	108	36
5	NMDARE	F	19	55	54
6	NMDARE	F	16	74	9
7	NMDARE	F	26	46	8
8	NMDARE	F	31	37	77
9	NMDARE	F	50	51	9
other AEs					
1	ADEM	F	22	44	12
2	Def AE	M	17	19	17
3	BBE	M	31	37	26
4	HE	F	49	54	64
5	Def AE	M	34	44	6
6	Def AE	F	18	33	25
7	ADEM	F	36	38	12
8	ADEM	M	71	108	26
9	LE	F	46	197	7
10	LE	F	53	59	54
11	ProAE	M	40	186	41
12	ProAE	M	45	42	4

ADEM: acute disseminated encephalomyelitis, BBE: Bickerstaff's brainstem encephalitis, Def AE: definite autoimmune encephalitis, HE: Hashimoto's encephalopathy, LE: limbic encephalitis, NMDARE: anti-N-methyl-d-aspartate receptor encephalitis, other AEs: other types of autoimmune encephalitis, ProAE: autoantibody-negative but probable autoimmune encephalitis

Supplementary Table 1. (continued)

case	prodrome	Abnormal behavior or cognitive dysfunction	speech dysfunction	seizure	Movement disorder	decreased level of consciousness	Autonomic dysfunction or central hypoventilation	fulfilled criteria for probable NMDARE
NMDARE								
1	+	+	+	+	+	+	+	+
2	-	+	+	+	+	+	+	+
3	+	+	+	+	+	+	+	+
4	+	+	+	+	+	+	-	+
5	-	+	+	+	-	-	-	-
6	+	+	+	-	+	+	+	+
7	+	+	+	-	-	+	-	-
8	+	+	+	-	-	-	-	-
9	+	+	+	+	+	-	-	+
other AEs								
1	+	+	-	+	-	+	+	+
2	+	+	-	+	-	-	-	-
3	-	+	-	-	-	-	+	-
4	+	+	+	+	-	+	-	+
5	+	+	-	-	-	+	+	-
6	+	+	+	-	-	+	+	+
7	+	+	-	-	-	+	+	-
8	-	+	-	-	-	+	-	-
9	+	+	+	-	+	+	+	+
10	-	+	+	+	-	+	+	+
11	+	+	-	-	-	+	+	-
12	+	-	-	-	-	+	+	-

NMDARE: anti-*N*-methyl-d-aspartate receptor-r encephalitis, other AEs: other types of autoimmune encephalitis, +: yes, -: no

Supplementary Table 1. (continued)

case	pleocytosis (cell >5 / μL)	antibodies				MRI abnormality
		In-house screening assays		confirmed antigen	others	
		TBA (pattern)	Live-neuron assay			
NMDARE						
1	+	Positive (neuropil)	Positive	NMDAR		+
2	+	Positive (neuropil)	Positive	NMDAR		-
3	+	Positive (neuropil)	Positive	NMDAR		-
4	+	Positive (neuropil and intracellular)	Positive	NMDAR, SOX1		-
5	-	Positive (neuropil)	Positive	NMDAR		-
6	+	Positive (neuropil)	Positive	NMDAR		-
7	+	Positive (neuropil)	Positive	NMDAR		-
8	+	Positive (neuropil)	Positive	NMDAR		-
9	+	Positive (neuropil)	Positive	NMDAR		+
other AEs						
1	+	Positive (astrocytic)	Negative	none		+
2	+	Positive (white matter)	Negative	MOG		+
3	+	Negative	Negative	none	GQ1b	-
4	-	Negative	Negative	none	TPO, Tg	+
5	+	Positive (neuropil)	Positive	none		-
6	+	Positive (neuropil)	Positive	none		-
7	+	Negative	Negative	none		+
8	-	Negative	Negative	none		+
9	+	Negative	Negative	none		+
10	-	Negative	Negative	none		+
11	+	Negative	Negative	none		+
12	+	Negative	Negative	none		+

CSF: cerebrospinal fluid, MOG: myelin oligodendrocyte glycoprotein, MRI: magnetic resonance imaging, NMDAR: *N*-methyl-d-aspartate receptor, NMDARE: anti-N-methyl-d-aspartate receptor encephalitis, other AEs: other types of autoimmune encephalitis, SOX1: SRY-Related HMG-Box Gene 1, TBA: Tissue-based assay, Tg: thyroglobulin, TPO: Thyroid peroxidase, +: yes, -: no

Supplementary Table 1. (continued)

case	EEG recording from onset (days)	EEG findings					
		Focal or diffuse slowing	beta activity[1]	epileptiform activity	EDB	RDA[2]	LPD
NMDARE							
1	7	+	+	-	+	-	-
2	5	+	+	-	-	+	-
3	9	+	+	-	-	+	-
4	8	+	-	-	-	+	-
5	2	+	-	+	-	-	-
6	23	+	+	-	-	-	-
7	10	+	-	-	-	-	-
8	12	+	-	-	-	-	-
9	2	+	+	-	-	-	-
other AEs							
1	21	+	-	+	-	+	-
2	1	+	-	-	-	+	-
3	15	+	-	-	-	-	-
4	2	+	+	-	-	-	-
5	21	+	-	-	-	+	-
6	4	+	-	-	-	+	-
7	17	+	-	-	-	+	-
8	10	+	-	-	-	-	-
9	32	+	-	-	-	+	-
10	1	+	-	-	-	-	+
11	10	+	-	-	-	-	-
12	13	+	-	-	-	+	-

EDB: extreme delta brush, EEG: electroencephalogram, LPD: lateralized periodic discharges, NMDARE: anti-*N*-methyl-d-aspartate receptor encephalitis; other AEs: other types of autoimmune encephalitis, RDA: rhythmic delta activity, +: detected, -: not detected

[1] Beta activity included diffuse or focal beta activity and excessive beta activity

[2] RDA included focal or generalized and intermittent or continuous RDA

Supplementary Table 1. (continued)

case	FSR at onset	FSR >cut off	FSR in the recovery phase	EEG recording in the recovery phase (months)
NMDARE				
1	0.070	+	0.412	58
2	0.048	+	0.270	37
3	0.057	+	0.213	29
4	0.283	+	0.444	31
5	0.139	+	0.221	28
6	0.771	+	N/A	N/A
7	0.109	+	0.259	18
8	0.341	+	0.436	12
9	0.588	+	N/A	N/A
other AEs				
1	0.035	-	0.644	6
2	0.046	-	0.197	10
3	0.247	+	N/A	N/A
4	0.357	+	0.355	34
5	0.086	+	N/A	N/A
6	0.024	-	0.117	39
7	0.040	-	N/A	N/A
8	0.045	-	0.460	3
9	0.020	-	0.208	6
10	0.023	-	0.400	65
11	0.018	-	N/A	N/A
12	0.010	-	N/A	N/A

EEG: electroencephalogram, FSR: fast slow ratio, NMDARE: anti-*N*-methyl-d-aspartate receptor encephalitis, N/A: not available; other AEs, other types of autoimmune encephalitis, +: yes, -: no

Supplementary Table 1. (continued)

case	Intractable epilepsy (AEDs≧3)	Sedative drug required	Immunotherapies				mRS	
			IVMP	IVIg	Plasma exchange	Second line immunotherapies	worst	latest
NMDARE								
1	+	+	+	+		+	5	3
2			+	+		+	5	3
3	+	+	+			+	5	3
4			+	+			5	4
5			+	+			5	0
6		+	+	+		+	5	2
7		+	+	+		+	5	4
8			+	+			5	1
9	+		+	+	+		1	1
other AEs								
1			+	+			5	1
2			+	+			2	0
3			+	+			5	2
4		+	+				5	3
5			+				4	3
6	+	+	+				5	2
7			+	+			4	1
8			+	+			5	4
9			+	+	+		5	4
10		+	+				5	4
11		+	+				5	4
12		+	+				5	4

AEDs: anti-epileptic drugs, IVMP: intravenous methylprednisolone, IVIg: intravenous immunoglobulins, mRS: modified Rankin scale, NMDARE: anti-*N*-methyl-d-aspartate receptor encephalitis, other AEs: other types of autoimmune encephalitis

4.2 Supplementary Table 2. Number and frequency of patients who met criteria of probable NMDARE and patients whose FSR was higher than our cutoff value

		higher FSR than cutoff		
		yes, n (%)	no, n (%)	total, n (%)
NMDARE group (n = 9)				
proNMDARE	yes, n (%)	6 (67)	0 (0)	6 (67)
	no, n (%)	3 (33)	0 (0)	3 (33)
	total, n (%)	9 (100)	0 (0)	9 (100)
other pAEs group (n = 14)				
proNMDARE	yes, n (%)	1 (7)	5 (36)	6 (43)
	no, n (%)	3 (21)	5 (36)	8 (57)
	total, n (%)	4 (28)	10 (72)	14 (100)

NMDARE: anti-N-methyl-d-aspartate receptor encephalitis, other pAEs: possible autoimmune encephalitis other than NMDARE, proNMDARE: probable NMDARE, FSR: fast slow ratio

5 Supplementary references

1. Hara M, Martinez-Hernandez E, Ariño H, Armangué T, Spatola M, Petit-Pedrol M, et al. Clinical and pathogenic significance of IgG, IgA, and IgM antibodies against the NMDA receptor. *Neurology* (2018) 90(16):e1386-e94. doi:10.1212/wnl.0000000000005329

2. Graus F, Titulaer MJ, Balu R, Benseler S, Bien CG, Cellucci T, et al. A clinical approach to diagnosis of autoimmune encephalitis. *Lancet Neurol* (2016) 15(4):391-404. doi:10.1016/s1474-4422(15)00401-9

3. Foff EP, Taplinger D, Suski J, Lopes MB, Quigg M. EEG Findings May Serve as a Potential Biomarker for Anti-NMDA Receptor Encephalitis. *Clin EEG Neurosci* (2017) 48(1):48-53. doi:10.1177/1550059416642660

4. Newson JJ, Thiagarajan TC. EEG Frequency Bands in Psychiatric Disorders: A Review of Resting State Studies. *Front Hum Neurosci* (2018) 12:521. doi:10.3389/fnhum.2018.00521

5. Benwell CSY, Davila-Pérez P, Fried PJ, Jones RN, Travison TG, Santarnecchi E, et al. EEG spectral power abnormalities and their relationship with cognitive dysfunction in patients with Alzheimer's disease and type 2 diabetes. *Neurobiol Aging* (2020) 85:83-95. doi:10.1016/j.neurobiolaging.2019.10.004

6. Molteni E, Avantaggiato P, Formica F, Pastore V, Colombo K, Galbiati S, et al. Sleep/Wake Modulation of Polysomnographic Patterns has Prognostic Value in Pediatric Unresponsive Wakefulness Syndrome. *J Clin Sleep Med* (2016) 12(8):1131-41. doi:10.5664/jcsm.6052
